# An fMRI-Neuronavigated Chronometric TMS Investigation of V5 and Intraparietal Cortex in Motion Driven Attention

**DOI:** 10.3389/fnhum.2017.00638

**Published:** 2018-01-04

**Authors:** Bonnie Alexander, Robin Laycock, David P. Crewther, Sheila G. Crewther

**Affiliations:** ^1^Murdoch Children’s Research Institute, Parkville, VIC, Australia; ^2^School of Psychology and Public Health, La Trobe University, Bundoora, VIC, Australia; ^3^School of Health and Biomedical Sciences, RMIT University, Melbourne, VIC, Australia; ^4^Centre for Human Psychopharmacology, Swinburne University, Hawthorn, VIC, Australia

**Keywords:** transcranial magnetic stimulation, intraparietal sulcus, motion processing, visual attention, functional chronometry, feedback

## Abstract

The timing of networked brain activity subserving motion driven attention in humans is currently unclear. Functional MRI (fMRI)-neuronavigated chronometric transcranial magnetic stimulation (TMS) was used to investigate critical times of parietal cortex involvement in motion driven attention. In particular, we were interested in the relative critical times for two intraparietal sulcus (IPS) sites in comparison to that previously identified for motion processing in area V5, and to explore potential earlier times of involvement. fMRI was used to individually localize V5 and middle and posterior intraparietal sulcus (mIPS; pIPS) areas active for a motion driven attention task, prior to TMS neuronavigation. Paired-pulse TMS was applied during performance of the same task at stimulus onset asynchronies (SOAs) ranging from 0 to 180 ms. There were no statistically significant decreases in performance accuracy for trials where TMS was applied to V5 at any SOA, though stimulation intensity was lower for this site than for the parietal sites. For TMS applied to mIPS, there was a trend toward a relative decrease in performance accuracy at the 150 ms SOA, as well as a relative increase at 180 ms. There was no statistically significant effect overall of TMS applied to pIPS, however, there appeared a potential trend toward a decrease in performance at the 0 ms SOA. Overall, these results provide some patterns of potential theoretical interest to follow up in future studies.

## Introduction

Speed of response to an oncoming obstacle is evolutionarily consequential. Thus research to better understand the neural mechanisms associated with motion driven attention networks in the human brain is theoretically important.

Seminal hierarchical models of the visual system derived from work in macaque established an initial feedforward sweep of information from retina through thalamus and superior colliculus, then via primary visual cortex (V1) on to a range of increasingly higher order areas including parietal and temporal cortex (Van Essen and Maunsell, 1983; Felleman and Van Essen, 1991; Van Essen et al., 1992). Retinofugal structural pathways to temporal (e.g., Sincich et al., 2004) and parietal cortex (e.g., Blatt et al., 1990; Boussaoud et al., 1990; Hardy and Lynch, 1992; Clower et al., 2001; Rushworth et al., 2006; Leh et al., 2008) that bypass V1 have also been described.

Subsequently, timing-based models of the visual system have posited recurrent feedback from higher-order processing areas (Lamme and Roelfsema, 2000; Bullier, 2001; Foxe and Simpson, 2002; Hochstein and Ahissar, 2002; Laycock et al., 2007b). In Bullier’s model, rapidly activated neurons with high conduction velocity axons and large receptive fields ‘retroinject’ approximate information about a visual scene onto neurons with small receptive fields, particularly those in V1 and V2, thereby influencing or focusing the processing in such lower order areas. This mechanism may be consistent with the concept of attentional salience mapping or gain control (Saalmann et al., 2007) such as is attributed to lateral intraparietal area (LIP) in macaque (Bisley and Goldberg, 2003; Gottlieb, 2007). The putative human homolog of LIP, middle IPS, is part of a large, extensively investigated network subserving attention (see reviews of attention networks, e.g., Corbetta and Shulman, 2002; Posner and Rothbart, 2007; Scolari et al., 2015). Investigating relative timing of LIP neurons in macaque, Saalmann et al. (2007) found that in the first 300 ms post-stimulus in a spatial attention task, activity in LIP could be shown to directly precede that in area MT in a percentage of trials. Ruff et al. (2008) demonstrated top-down influence from parietal cortex in humans with concurrent transcranial magnetic stimulation-functional MRI (TMS-fMRI), finding that stimulation of parietal cortex led to BOLD activity changes in V1.

In recent years there has been increased emphasis on the role of thalamic nuclei in attentional regulation. Saalmann et al. (2012) suggested that macaque pulvinar nucleus synchronizes cortical activity, depending on the locus of attention, and that visual processing models be reconsidered to include pulvino-cortical feedback at each stage of cortical involvement. Within such a complex hierarchical network, there seems likely an abundance of candidate locales or subcircuits via which feedforward and feedback activity might occur. The current paper focuses on critical times of intraparietal sulcus (IPS) and V5 involvement in motion driven attention, while acknowledging the context of the complex systems in which these areas are functionally situated.

Transcranial magnetic stimulation has been employed in a chronometric mode to investigate the necessary timing of activity in visual processing networks. Although differential timing of behavioral effects of TMS at two sites cannot facilitate inference of direct structural connection or conduction velocity (Scharnowski et al., 2013), there are arguably situations in which such timing data may provide clues to network dynamics. For example, if the initial sweep of visual activity includes a more direct route to parietal areas than that passing through the more traditional geniculostriate pathway, this could be indicated by activity in parietal areas earlier than that seen in V1. The robustly demonstrated ‘classical dip’ in behavioral performance for TMS applied to V1 occurs around 100 ms after stimulus onset (de Graaf et al., 2014). However, there are earlier time points for which TMS-induced visual masking has been reported such as a ∼30 ms dip, though less reliably (Corthout et al., 1999; Laycock et al., 2007a; de Graaf et al., 2014).

Regarding the effects of TMS on motion perception specifically, Laycock et al. (2007a) found critical times of area V1/V2 involvement in motion direction discrimination at 0 and 125 ms after motion onset, and area V5/MT+ involvement at 0–30 and 158 ms. Critical periods for V5 in motion processing have also been reported in other chronometric TMS studies at similarly early times (Beckers and Homberg, 1992; Beckers and Zeki, 1995; Walsh et al., 1998; d’Alfonso et al., 2002), and around 150 ms (e.g., Hotson et al., 1994; Anand et al., 1998; Hotson and Anand, 1999; d’Alfonso et al., 2002; Sack et al., 2006; Stevens et al., 2009). Laycock et al. (2007a) suggest that this latter period may reflect processing associated with feedback signals from higher order frontal or parietal areas. The timing of involvement of IPS in motion processing does not appear to have been previously reported with chronometric TMS.

The current experiment investigated the timing of necessary involvement of IPS and V5 in motion driven attention. First, fMRI was used to find BOLD activity subserving rapid, motion driven attention, to be used for individually localized TMS application. To ensure that areas to be stimulated were not simply subserving eye movements alone, this included delineation at a group level of activity distinct from eye movement-related parietal activation. Neuronavigated TMS was then employed to measure times of necessary involvement of two identified parietal sites, and V5 (also localized with fMRI), in motion driven attention. Necessary involvement was indicated by decreased behavioral accuracy in performing a motion driven attention task with TMS delivered at particular stimulus onset asychronies (SOAs). The aim was to establish the temporal relationship between critical involvement of parietal regions and V5 around 150 ms, and also to explore potentially earlier times of involvement of IPS in motion driven attention.

## Materials and Methods

### Participants

Four women and seven men (18–35 years; *M* = 28.90, *SD* = 3.18) participated in fMRI testing. The sample was a convenience sample of people known by the authors and individuals attending La Trobe University, and all were university educated. Inclusion criteria required normal or corrected to normal vision, no history of neurological or psychiatric disease; and for safety purposes, no metal implants, or family history of epilepsy or seizures. Nine of the initial 11 participants (three women, six men, age *M* = 28.5, *SD* = 3.38) proceeded to TMS testing. Of these, one woman and one man were left-handed.

### fMRI Localisation of Intraparietal Activity for Motion Driven Attention

Functional MRI localisation utilized a novel Motion Driven Attention task (designed here and also employed in Wang et al., 2017) designed to elicit activity in areas including IPS and V5 at a group level. A previously used saccade generation task (Sestieri et al., 2008) was used for comparison to clarify that the parietal areas to be stimulated with TMS were not representative solely of saccade production, considering possible nearby involvement of ‘parietal eye fields’ (PEFs; Müri et al., 1996; Thier and Andersen, 1998; Goldberg et al., 2002). BOLD activity was defined on an individual basis for neuronavigation, both for the Motion Driven Attention task, and for a standard V5 localizer task.

### fMRI Tasks

#### Motion Driven Attention Task

**FIGURE 1:**
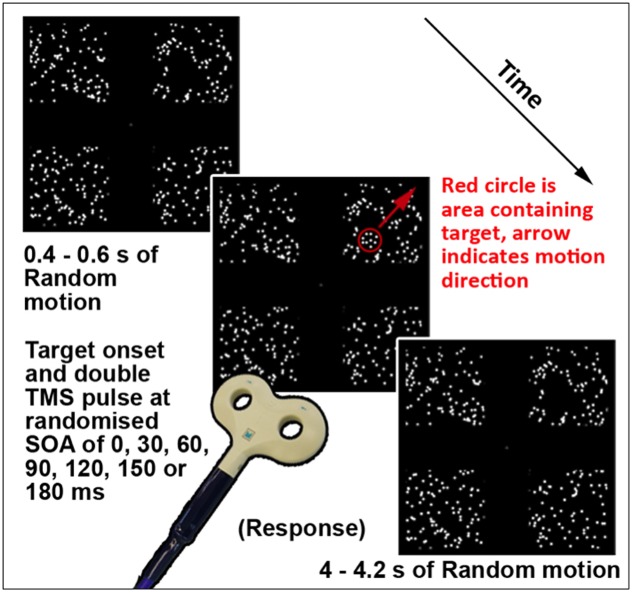
Schematic of a single trial of the TMS version of Motion Driven Attention task. The red circle and arrow are for schematic purposes only.

This task (TMS version illustrated in **Figure 1**; see Section “Chronometric fMRI-Neuronavigated TMS”) was created and displayed using VPixx software and a DATAPixx unit (VPixx Technologies, Inc., Saint-Bruno-de-Montarville, QC, Canada). It featured a coherent motion target amidst a background of random motion, presented in one of four square regions displayed in the top left, top right, bottom left, and bottom right quadrants of the screen. Each region subtended a visual angle of 14° × 14°, with 0.46 dots per square degree. Randomly moving white dots on a black background were presented continually in each region. The dots were 0.50° in diameter, moved at 3°/s, and had a lifetime of six frames or 100 ms. A 60 Hz refresh rate was used, thus each frame was 16.67 ms. The target was a smaller form defined by coherently moving dots inside a circular region. The dots in the target were the same size, density, and brightness as the surrounding randomly moving dots. The target moved diagonally outward, from the inner to the outer corner of the randomly assigned quadrant per trial at 156°/s. The size of the target was adjusted between 4 and 8° diameter to achieve required accuracy levels per participant, as the target was more easily perceptible with increasing size. A gray central crosshair was present throughout the task. On each trial, the randomly moving dots were first presented for between 400 and 600 ms and remained on the screen for the whole trial. Subsequently the target was presented in one pseudo-randomized quadrant for 116.67 ms, or seven frames. For this fMRI version of this task, each trial was 4000 ms in total, as necessitated by scanner-related requirements. The TMS version featured variable length trials, in order to maximize the unpredictability of onset of the target stimulus. This version is described further in Section “Chronometric fMRI-Neuronavigated TMS.” Participants were asked to focus on the fixation crosshair, and indicate via button press which quadrant the stimulus appeared in, if it was perceived. Forced choice guesses were required for trials where a target stimulus was not perceived. The version of this task used during fMRI was a block design with four active (target presented) and four baseline blocks (target absent). Each block ran for eight volumes, or 24 s. Instructions were presented between each block for 4 s, and the task ran for 252 s. During baseline blocks, participants were asked to press any key when a new trial began, indicated by the crosshair briefly disappearing and returning.

#### Saccade Task

This block design task was based on that utilized by Sestieri et al. (2008) to elicit saccade-related BOLD activity. Six 30 s baseline blocks alternated with five 30 s active blocks. In the active condition, white squares subtending 2.18° visual angle appeared for 500 ms in one of five alternating locations: centrally, and at 6 and 12° to the left and right of center. The baseline condition was a fixation crosshair. Participants were asked to look at each appearing square during the active condition, and to focus on the crosshair during baseline.

#### V5 Localizer

A standard V5 localizer task was used, featuring radially moving dots contrasted with stationary dots (as per, e.g., Tootell et al., 1995; Sack et al., 2006). A block design alternated six 15 s blocks of radial motion with six blocks featuring stationary white dots in a circular region on a black background. This region subtended 25° visual angle, with 0.5 dots per square degree. Each dot was 0.36° diameter. In the motion condition the dots repeatedly moved radially inward for 2.5 s and outward for 2.5 s, with 100% coherence, at 20°/s measured at 15° from the center. This task was passively viewed, and participants were asked to focus on a central crosshair.

### MRI Acquisition

Scanning was conducted at the Brain Research Institute, Austin Repatriation Hospital, Heidelberg, Melbourne, Australia, on a 3T Siemens Trio Tim scanner. Participants viewed a 44 cm × 27 cm LCD monitor at 2.5 m distance via a mirror mounted in the head coil, and responded via a four-button response box. Functional images were acquired with: 44 axial slices, 3 mm isotropic voxels, TR = 3000 ms, TE = 30 ms, gap thickness = 3 mm, FOV = 216 mm, flip angle = 85°, interslice time = 68 ms. T1 weighed structural images were acquired using: 44 axial slices, 0.9 mm isotropic voxels, TR = 1900 ms, TE = 2.6 ms, gap thickness = 3 mm, flip angle = 9°.

### Preprocessing and Analysis

Preprocessing and analysis was performed using BrainVoyager QX 64 v2.2 (Brain Innovation, Maastricht, Netherlands). T1-weighted images were resampled to 1 mm isotropic voxels, AC-PC aligned, and transformed into Talairach space (Talairach and Tournoux, 1988) for group analyses. Head surface and right hemisphere cortical surface reconstructions were generated from the T1 images for TMS neuronavigation.

Functional MRI preprocessing comprised: 3D motion correction, slice scan time correction, spatial smoothing with a 3 mm Full Width Half Maximum kernel to reduce noise but maintain resolution for localisation of areas of interest per individual (as per Sack et al., 2008), and temporal high pass filtering using a Fourier basis set with linear trend removal, with a cutoff value of five cycles per time-course. Functional data were coregistered with structural scans in AC-PC space for individual analyses and TMS application, and in Talairach space for group analyses.

For both the motion driven attention task and the saccade task, a random effects group GLM analysis was performed using *N* = 11 participants, and a contrast subtracting baseline from active conditions. The *t*-map was thresholded at *p* < 0.01, and for both tasks voxelwise cluster extent thresholding using α < 0.05 determined a minimum cluster size *k* = 8, used for multiple comparison correction. A random effects group GLM analysis was performed for the V5 task, thresholded at *p* < 0.005, and a contrast subtracting activity for the baseline condition from the motion condition. Cluster extent thresholding with α < 0.05 determined a minimum cluster size of *k* = 6.

### fMRI Results and Region of Interest Selection

The Motion Driven Attention task elicited activity at the group level in IPS (middle and posterior) and V5, as well as frontal eye fields (FEFs) and parieto-occipital areas including Middle Occipital Gyrus and Superior Parieto-Occipital Cortex, and bilateral insulae. Parietal activity was stronger and more extensive in the right hemisphere. See Supplementary Table S1 for cluster information. The Saccade task confirmed activity in areas similar to those in Sestieri et al. (2008), including FEF, right-lateralized PEF/IPS, and V5 (see Supplementary Table S2 for cluster details). The V5 task showed focused bilateral temporal activity (right hemisphere Talairach coordinate *x* = 50, *y* = -71, *z* = 3) consistent with V5 as defined in the literature (e.g., Watson et al., 1993; Tootell et al., 1995; Dumoulin et al., 2000; Rees et al., 2000), and bilateral activity in occipital areas consistent with early visual system.

**FIGURE 2:**
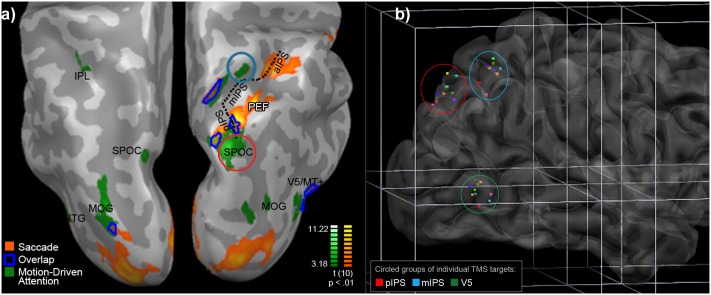
**(a)** Motion Driven Attention (green) and Saccade task (orange) positive activity, and overlap (dark blue outline), from RFX analyses (*N* = 11, *p* < 0.01, *k* = 8). Displayed on inflated cortices of one participant, in neurological orientation from above the parietal area. PEFs, parietal eye fields; IPL, inferior parietal lobule; SPOC, superior parieto-occipital cortex; MOG, middle occipital gyrus; ITG, inferior temporal gyrus; V5/MT, visual area 5/middle temporal; aIPS, mIPS, pIPS, inferior, middle, and posterior Intraparietal Sulcus, respectively. **(b)** Single points for TMS application per individual in Talairach space. Transparency is used to display points in sulci or below the surface. Green, red, and blue ellipses contain one point for each participant for the areas indicated. Consistent colors of points are used across areas per participant.

Localisation of IPS for TMS included seeking areas where the Motion Driven Attention task elicited activity separable from that for the Saccade task. Overlap for these tasks (see **Figure 2**) included FEF, bilateral V5/MT+, areas in right middle and posterior parietal cortex, including a section of the medial bank of the middle section of IPS. Anterolateral to this was a cluster active for the Motion Driven Attention task only, at the threshold chosen (circled in blue in **Figure 2**). This cluster was chosen as a target region for TMS, hereafter termed ‘mIPS’ for brevity. Also overlapping were medial and lateral regions in the mid-posterior section of IPS. The Motion Driven Attention task yielded activity posterior to this, including a strong maximum of activity in posterior IPS extending to Superior Parieto-Occipital Cortex (circled in red in **Figure 2**). This area was also chosen for TMS application, and termed ‘pIPS.’

Individual fMRI localisation is described in Supplementary Information Section D1. Selection of TMS targets in the chosen regions of interest took into account both group activity and local individual maxima (see Supplementary Table S3). The mean (SD in brackets) Talairach coordinates (x, y, z) stimulated were: mIPS: 26.6(2.0), -60.1(2.4), 51.7(4.6); pIPS: 14.7(4.2), -77.7(3.8), 42.3(4.1); V5: 45.2(4.7), -69.6(2.4), 2.4(3.7). Individual points for each target region are illustrated in **Figure 2b**.

### Chronometric fMRI-Neuronavigated TMS

Transcranial magnetic stimulation was used to determine critical timing of activation of V5, mIPS, and pIPS throughout the first 180 ms after visual target onset in the motion driven attention task.

The version of this task used for TMS testing (illustrated in **Figure 1**) featured the same display parameters as that used for fMRI testing, described in Section “Motion Driven Attention Task.” The timing per trial was modified to include TMS triggers and a randomized trial ending length of between 4 and 4.2 s after the target stimulus onset, meaning each trial lasted between 4.4 and 4.8 s. In terms of overall task structure, the version of this task during which TMS was applied contained no component baseline condition or trials, hence each trial contained a target stimulus and triggered TMS. A thresholding version of the task was performed using various sizes of the target stimulus, to redetermine performance thresholds. Using the established parameters for the thresholded level, a short, 32 trial ‘No-TMS’ version task was run again to validate threshold levels and equal performance levels for visual quadrants in absence of TMS.

#### Protocol and Procedure

Right hemisphere mIPS, pIPS, and V5 were stimulated in separate blocks in counterbalanced order across participants, and served as control sites for each other (Sandrini et al., 2011). A No-TMS condition was used to ensure accuracy levels and consistent performance between quadrants of stimulus presentation in absence of TMS. Each block was approximately 9 min. Each trial had a randomized TMS SOA of 0, 30, 60, 90, 120, 150, or 180 ms time-locked to the motion target onset, and randomized quadrant of stimulus presentation. Paired monophasic pulses were used with a 5 ms interstimulus interval. Maximum stimulation intensity was 60% stimulator output. The coil was angled with the handle pointing backward for all sites. TMS was performed with a Magstim Bistim^2^ machine, interchangeable Magstim Double 70 mm coils, and a Magstim Articulated Coil Stand (The Magstim Company, Ltd., Wales, United Kingdom), and triggered via a DATAPixx unit. Responses were given via a RESPONSEPixx response box (VPixx Technologies, Inc., Saint-Bruno-de-Montarville, QC, Canada). Neuronavigation was performed using BrainVoyager QX 2.1 TMS Neuronavigator software (Brain Innovation, Maastricht, Netherlands). A Zebris CMS20 ultrasound-based system (Zebris Medical GmbH, Isny, Germany) was used for head and coil registration and monitoring. The motion driven attention task was displayed on a 32 inch LCD monitor, at viewing distance of 171 cm. Individual accuracy was first thresholded to approximately 70%. The task was then performed with no TMS. Coregistration, TMS intensity level establishment and navigation were then performed. Intensity was gradually increased toward 60%, and was reduced if the participant indicated discomfort. Average stimulation intensity was 57.89% for mIPS, 59.11% for pIPS, and 45.56% for V5. These procedures are described in detail in Supplementary Information Section D2. TMS was performed with continual neuronavigation through each block.

#### Data Handling and Analysis

Transcranial magnetic stimulation data were normally distributed, and outliers were corrected, for both accuracy and reaction time measures. The procedure for outlier correction was as follows: data for all variables were screened for outliers. Across all accuracy variables, 2.2% (five values) of data points were identified as lying outside the upper or lower limit, defined as the 3rd quartile plus 1.5 × the interquartile range, or the 1st quartile minus 1.5 × the interquartile range, respectively. Of these values, one was identified as an extreme value, i.e., the 3rd quartile plus 3 × the interquartile range or the 1st quartile minus 3 × the interquartile range. For reaction time data, 6.3% (12 values) of values were identified as outliers, four of which were extreme values. Notably, the data-points corresponded to different participants per site of TMS. Considering that extreme values were not consistent across the entire experiment, or specific to one participant, it was decided to retain all participants’ data and correct extreme values (e.g., Tabachnick and Fidell, 2014). Outliers were corrected to the values of the upper or lower limit. This limit was chosen to conserve power by retaining the sample size, while also preserving the patterns of variation in the data. Shapiro–Wilk tests were performed for accuracy and reaction time, for each combination of site and SOA, and data were found to be normally distributed. Z-scores of skew and kurtosis (used to indicate deviations from normality for small sample sizes; see Tabachnik and Fidell) were also computed and were found to be well within the required range for normality between -1.96 and 1.96. Results of Shapiro–Wilk tests, and z-skew and z-kurtosis data, are included in Supplementary Tables S6, S7.

For each condition, there was considerable variability between participants in the average accuracy level across SOAs. As the construct of interest was relative timing of changes in performance, rather than overall accuracy pooled across SOAs, data were normalized based on each individual’s average accuracy across all SOAs. Normalization was performed for each cortical site separately. All data, including raw and normalized scores and means, are provided in Supplementary Data.

One way ANOVAs were used to test for relative differences in performance across all SOAs, with η^2^ indicating effect size. Where assumptions of sphericity were not met, Huynh-Feldt corrected values are reported. *Post hoc* two-tailed single sample *t*-tests and Cohen’s *d* (Cohen, 1988) were used to clarify differences between mean accuracy *z*-scores and zero (i.e., the average normalized performance across all SOAs) for specific SOAs. Results are discussed with an emphasis on effect size (using Cohen’s *d*, where 0.2 is small, 0.5 is medium, and 0.8 indicates a large effect), rather than using *p*-values as a dichotomous decision criterion (see Cumming, 2012). *Post hoc* single sample *t*-tests were used to test the difference between accuracy z-score and 0, for each SOA. These were uncorrected for multiple comparisons (as per, e.g., de Graaf et al., 2011). Though this lack of correction is suboptimal, it is necessary given the numerous SOAs, conditions, and small sample size.

Reaction time analysis was also performed, though considered of secondary importance, and thus for brevity is included as Supplementary Figure S1 and Supplementary Table S5, and following text. Results for accuracy are presented below.

## TMS Results

**FIGURE 3:**
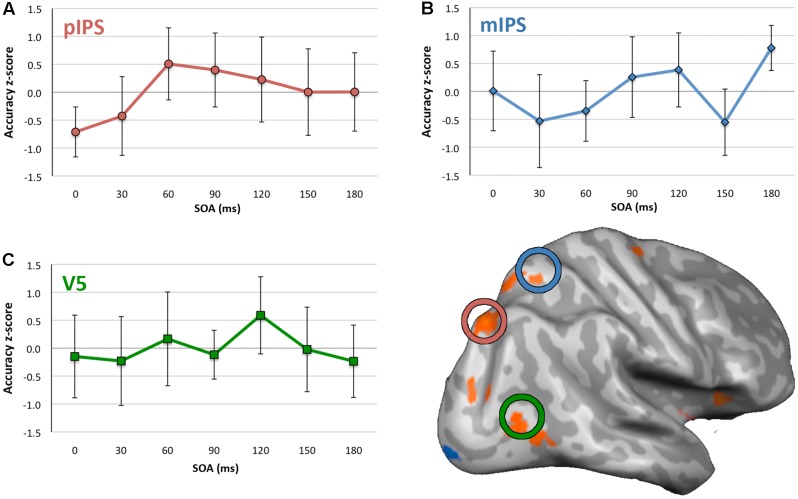
Mean accuracy and 95% confidence intervals for each SOA for each site of stimulation. **(A)** pIPS, **(B)** mIPS, and **(C)** V5. Y axes show accuracy z-scores, x axes are SOA between 0 and 180 ms. Inflated right hemisphere surface shown with group activity for the Motion Driven Attention task, and mIPS, pIPS, and V5 TMS application sites circled. N.B., confidence intervals pertain to the difference between accuracy z-score and 0, at each SOA. They do not indicate significance of accuracy differences across SOAs. Where the intervals exclude 0, this indicates statistically significant difference from 0.

For the no-TMS condition, ANOVA indicated no significant difference in accuracy between quadrants, *F*(3,24) = 1.385, *p* = 0.271, η^2^ = 0.148. For V5 TMS, ANOVA revealed no statistically significant effect of SOA on accuracy (**Figure 3C**), *F*(6,48) = 0.776, *p* = 0.592, η^2^ = 0.088. For mIPS TMS, there was a significant effect of SOA with a large effect size, *F*(6,48) = 2.724, *p* = 0.023, η^2^ = 0.255 (**Figure 3B**). *Post hoc t*-tests (see Supplementary Table S4) found a significant increase at the 180 ms SOA with a very large effect size (*t*(8) = 4.452, *p* = 0.002, *d* = 1.484, 95%CI[0.376, 1.183]). The trend toward a decrease at the 150 ms SOA was not significant, however did have a medium to large effect size, *t*(8) = -2.140, *p* = 0.065, *d* = 0.713, CI[-1.145, 0.043]. For pIPS TMS, ANOVA revealed no statistically significant effect of SOA, *F*(6,48) = 1.907, *p* = 0.099, η^2^ = 0.193, however the effect size here was medium to large.

## Discussion

The functional chronometry of areas V5, mIPS, and pIPS in motion driven attention was investigated using neuronavigated TMS and shown not to be significantly different at any SOA below 180 ms in any area. A significant increase in accuracy was seen following TMS at 180 ms post the onset of the motion driven attention stimulus in mIPS after a non-significant trend toward a decrease in performance at the 150 ms SOA. Accuracy on the No-TMS condition of the Motion Driven Attention task was shown to be not significantly different when compared across visual quadrants, indicating that the task *per se* did not elicit spatially biased performance.

There was no difference in accuracy of performance on our Motion Driven Attention task for any SOA for TMS applied to V5. In other studies, comparable TMS to V5 around 0 or 30 ms has been reported to inhibit motion direction discrimination (Beckers and Homberg, 1992; Beckers and Zeki, 1995; Laycock et al., 2007a) and lead to a performance drop at 150 ms for motion-based tasks (e.g., Hotson et al., 1994; Hotson and Anand, 1999; Sack et al., 2006; Laycock et al., 2007a; Stevens et al., 2009). Thus, either the times of critical involvement in motion processing and motion driven attention are not comparable, or the intensity of the stimulation or other parameters were not adequate. Confounding factors here were lower stimulation intensity (around 45% stimulator output) than for parietal ROIs (near 60%) due to participant discomfort, in some cases minor muscle twitches (see Supplementary Information Section D1), and presumably greater distraction. As V5 is close to the cervical and trigeminal nerves, it seems possible this may be common in TMS studies involving V5. However, Laycock et al. (2007a) stimulated this area using a similar experimental setup and the same interpulse interval, at 60% stimulator output, and reported no such physiological issues. A related concern is that for all sites, some fMRI activity maxima per individual were below the surface of the brain in the wall of the sulcus rather than the bank. Thus, neural activity is disrupted by TMS in overlying gyri as well as in the identified sulcal targets.

For mIPS TMS, the significant increase in accuracy found at the 180 ms SOA was not anticipated. Given all data were normalized across all SOAs, this increase in accuracy may reflect a relative difference, rather an absolute increase in accuracy. Similarly, given that accuracy scores were normalized to an average across all SOAs, it could also be the case that all other SOAs showed a relative reduction in performance, rather than reflecting ‘enhanced’ performance at 180 SOA *per se*.

With regards to the literature regarding the timing of visual motion processing, the potential involvement of mIPS at 150 ms SOA was of theoretical interest, as a decrease at this timepoint may have indicated close involvement of this area and V5 in motion driven attention. However, the medium to large effect size for decreased average accuracy seen at the 150 ms SOA was not statistically significant, with the confidence interval slightly crossing zero for this point in **Figure 3B**. This may be considered marginal evidence that mIPS could potentially be relevant to motion driven attention at this time, and could be an indication that this is a candidate area of interest to follow up in future studies.

Similarly, for pIPS TMS, there appeared to be lower accuracy for the 0 ms SOA (indicated by confidence interval not overlapping zero in **Figure 3A**). However, though indicating a medium to large effect size, the ANOVA was not statistically significant. Were a significant decrease in accuracy found at this SOA, this could indicate prestimulus involvement of this area in motion driven attention. This potential early activation of pIPS also may be of interest to investigate further in future. However, as results for this small cohort stand it is not possible to make any definitive statement about when pIPS and mIPS areas of parietal cortex are critically involved in motion driven attention.

### Limitations

Some of the limitations of the current experiment relate to trade offs made in the TMS protocol design between the number of sites, SOAs and trials that were practical to include in testing sessions. Possibly the most salient among these was the restricted number of SOAs, which limited the temporal resolution and ability to test whether, for example, mIPS involvement in motion driven attention may have been critical at finer timepoints before and after 150 ms. This limited the comparisons that could be made between critical involvement of this area and the timing of involvement of V5 as reported in other chronometric studies in the literature.

The confounds in stimulating V5 relating to comfort levels and muscle twitches restricted the conclusions that could be made about the relative chronometry of this area in motion driven attention. A related concern is that for V5 and IPS ROIs, some of the fMRI activity maxima identified per individual were below the surface of the brain in the wall of the sulcus rather than the bank. This means that neural activity is induced by TMS in overlying or partially overlying gyri as well as in the identified sulcal targets. This may also be a systemic issue with neuronavigated TMS, however neuronavigated TMS has been clearly demonstrated to offer more power and accuracy than other methods of localisation (Sack et al., 2008), so this was not considered a major limitation.

Lastly, as mentioned earlier, the small sample size here, although typical of most traditional TMS studies, conferred low statistical power. The sample size was restricted due to prohibitive cost of incorporating any further MRI scanning. However, fMRI localisation greatly increases statistical power of TMS studies, compared with other localisation techniques (Sack et al., 2008). Therefore, we consider that we have conserved statistical power via use of individual fMRI neuronavigation, and importantly, we have gained precision and validity. fMRI localisation was also particularly critical to the validity of this study considering that IPS does not give rise to overt, readily-observable phenomena like phosphenes or motor output when stimulated. In future, large-scale cohort studies incorporating fMRI may provide fruitful circumstances in which to recruit participants for higher-powered TMS studies.

## Conclusion

This study investigated the timing of motion driven attention in areas V5 and middle and posterior IPS in healthy humans using individually fMRI-neuronavigated TMS. No statistically significant effects on performance on a motion driven attention task were detected for TMS applied to V5 or pIPS. For pIPS, there appeared to be a potential decrease at 0 ms, which could be a theoretically interesting pattern to note and potentially compare with results of future studies. An overall effect of TMS on mIPS was found, with a significant increase in accuracy at the 180 ms SOA, and a non-significant trend toward a decrease in performance at the 150 ms SOA. These results provide patterns of interest to potentially follow up in higher powered studies in future.

## Ethics Statement

This study was approved by and carried out in accordance with the recommendations of La Trobe University Human Ethics Committee and Swinburne University Human Research Ethics Committee with written informed consent from all subjects. All subjects gave written informed consent in accordance with the Declaration of Helsinki.

## Author Contributions

BA contributed to experimental design, created cognitive tasks, carried out MRI and TMS testing and data analysis, and prepared the manuscript. RL contributed to experimental design, carried out TMS testing, and revised the manuscript. SC initiated and contributed to the experimental design, and revised the manuscript. DC contributed to experimental design, provided access to TMS testing equipment and facilities, and revised the manuscript.

## Conflict of Interest Statement

The authors declare that the research was conducted in the absence of any commercial or financial relationships that could be construed as a potential conflict of interest.

## References

[B1] Anand S. Olson J. D. Hotson J. R. (1998). Tracing the timing of human analysis of motion and chromatic signals from occipital to temporo-parieto-occipital cortex: a transcranial magnetic stimulation study. *Vision Res.* 38 2619–2627. 10.1016/S0042-6989(98)00025-X 12116707

[B2] Beckers G. Homberg V. (1992). Cerebral visual motion blindness: transitory akinetopsia induced by transcranial magnetic stimulation of human area V5. *Proc. R. Soc. Lond. B Biol. Sci.* 249 173–178. 10.1098/rspb.1992.0100 1360678

[B3] Beckers G. Zeki S. (1995). The consequences of inactivating areas V1 and V5 on visual motion perception. *Brain* 118 49–60. 10.1093/brain/118.1.49 7895014

[B4] Bisley J. W. Goldberg M. E. (2003). Neuronal activity in the lateral intraparietal area and spatial attention. *Science* 299 81–86. 10.1126/science.1077395 12511644

[B5] Blatt G. J. Andersen R. A. Stoner G. R. (1990). Visual receptive field organization and cortico-cortical connections of the lateral intraparietal area (area LIP) in the macaque. *J. Comp. Neurol.* 299 421–445. 10.1002/cne.902990404 2243159

[B6] Boussaoud D. Ungerleider L. G. Desimone R. (1990). Pathways for motion analysis: cortical connections of the medial superior temporal and fundus of the superior temporal visual areas in the macaque. *J. Comp. Neurol.* 296 462–495. 10.1002/cne.902960311 2358548

[B7] Bullier J. (2001). Integrated model of visual processing. *Brain Res. Rev.* 36 96–107. 10.1016/S0165-0173(01)00085-6 11690606

[B8] Clower D. M. West R. A. Lynch J. C. Strick P. L. (2001). The inferior parietal lobule is the target of output from the superior colliculus, hippocampus, and cerebellum. *J. Neurosci.* 21 6283–6291. 11487651 10.1523/JNEUROSCI.21-16-06283.2001 PMC6763148

[B9] Cohen J. (1988). *Statistical Power Analysis for the Behavioral Sciences*, 2nd Edn Mahwah, NJ: Lawrence Erlbaum.

[B10] Corbetta M. Shulman G. L. (2002). Control of goal-directed and stimulus-driven attention in the brain. *Nat. Rev. Neurosci.* 3 201–215. 10.1038/nrn755 11994752

[B11] Corthout E. Uttl B. Ziemann U. Cowey A. Hallett M. (1999). Two periods of processing in the (circum) striate visual cortex as revealed by transcranial magnetic stimulation. *Neuropsychologia* 37 137–145. 10.1016/S0028-3932(98)00088-8 10080371

[B12] Cumming G. (2012). *Understanding the New Statistics: Effect Sizes, Confidence Intervals, and Meta-analysis.* New York, NY: Routledge.

[B13] d’Alfonso A. Van Honk J. Schutter D. Caffe A. Postma A. de Haan E. (2002). Spatial and temporal characteristics of visual motion perception involving V5 visual cortex. *Neurol. Res.* 24 266–270. 10.1179/016164102101199891 11958420

[B14] de Graaf T. A. Herring J. Sack A. T. (2011). A chronometric exploration of high-resolution ‘sensitive TMS masking’ effects on subjective and objective measures of vision. *Exp. Brain Res.* 209 19–27. 10.1007/s00221-010-2512-z 21161191 PMC3035793

[B15] de Graaf T. A. Koivisto M. Jacobs C. Sack A. T. (2014). The chronometry of visual perception: review of occipital TMS masking studies. *Neurosci. Biobehav. Rev.* 45 295–304. 10.1016/j.neubiorev.2014.06.017 25010557

[B16] Dumoulin S. O. Bittar R. G. Kabani N. J. Baker C. L. Jr. Le Goualher G. Pike G. B. (2000). A new anatomical landmark for reliable identification of human area V5/MT: a quantitative analysis of sulcal patterning. *Cereb. Cortex* 10 454–463. 10.1093/cercor/10.5.454 10847595

[B17] Felleman D. J. Van Essen D. C. (1991). Distributed hierarchical processing in the primate visual cortex. *Cereb. Cortex* 1 1–47. 10.1093/cercor/1.1.1 1822724

[B18] Foxe J. Simpson G. (2002). Flow of activation from V1 to frontal cortex in humans. *Exp. Brain Res.* 142 139–150. 10.1007/s00221-001-0906-7 11797091

[B19] Goldberg M. E. Bisley J. Powell K. D. Gottlieb J. Kusunoki M. (2002). The role of the lateral intraparietal area of the monkey in the generation of saccades and visuospatial attention. *Ann. N. Y. Acad. Sci.* 956 205–215. 10.1111/j.1749-6632.2002.tb02820.x 11960805

[B20] Gottlieb J. (2007). From thought to action: the parietal cortex as a bridge between perception, action and cognition. *Neuron* 53 9–16. 10.1016/j.neuron.2006.12.009 17196526

[B21] Hardy S. G. P. Lynch J. C. (1992). The spatial distribution of pulvinar neurons that project to two subregions of the inferior parietal lobule in the macaque. *Cereb. Cortex* 2 217–230. 10.1093/cercor/2.3.217 1511222

[B22] Hochstein S. Ahissar M. (2002). View from the top: hierarchies and reverse hierarchies in the visual system. *Neuron* 36 791–804. 10.1016/S0896-6273(02)01091-7 12467584

[B23] Hotson J. Anand S. (1999). The selectivity and timing of motion processing in human temporo-parieto-occipital and occipital cortex: a transcranial magnetic stimulation study. *Neuropsychologia* 37 169–179. 10.1016/S0028-3932(98)00091-8 10080374

[B24] Hotson J. Braun D. Herzberg W. Boman D. (1994). Transcranial magnetic stimulation of extrastriate cortex degrades human motion direction discrimination. *Vision Res.* 34 2115–2123. 10.1016/0042-6989(94)90321-2 7941409

[B25] Lamme V. A. F. Roelfsema P. R. (2000). The distinct modes of vision offered by feedforward and recurrent processing. *Trends Neurosci.* 23 571–579. 10.1016/S0166-2236(00)01657-X 11074267

[B26] Laycock R. Crewther D. P. Fitzgerald P. Crewther S. G. (2007a). Evidence for fast signals and later processing in human V1/V2 and V5/MT+: a TMS study of motion perception. *J. Neurophysiol.* 98 1253–1262. 17634339 10.1152/jn.00416.2007

[B27] Laycock R. Crewther S. G. Crewther D. P. (2007b). A role for the ‘magnocellular advantage’ in visual impairments in neurodevelopmental and psychiatric disorders. *Neurosci. Biobehav. Rev.* 31 363–376. 17141311 10.1016/j.neubiorev.2006.10.003

[B28] Leh S. E. Chakravarty M. M. Ptito A. (2008). The connectivity of the human pulvinar: a diffusion tensor imaging tractography study. *Int. J. Biomed. Imaging* 2008:789539. 10.1155/2008/789539 18274667 PMC2233985

[B29] Müri R. M. Iba-Zizen M. T. Derosier C. Cabanis E. A. Pierrot-Deseilligny C. (1996). Location of the human posterior eye field with functional magnetic resonance imaging. *J. Neurol. Neurosurg. Psychiatry* 60 445–448. 10.1136/jnnp.60.4.445 8774415 PMC1073903

[B30] Posner M. I. Rothbart M. K. (2007). Research on attention networks as a model for the integration of psychological science. *Annu. Rev. Psychol.* 58 1–23. 10.1146/annurev.psych.58.110405.085516 17029565

[B31] Rees G. Friston K. Koch C. (2000). A direct quantitative relationship between the functional properties of human and macaque V5. *Nat. Neurosci.* 3 716–723. 10.1038/76673 10862705

[B32] Ruff C. C. Bestmann S. Blankenburg F. Bjoertomt O. Josephs O. Weiskopf N. (2008). Distinct causal influences of parietal versus frontal areas on human visual cortex: evidence from concurrent TMS-fMRI. *Cereb. Cortex* 18 817–827. 10.1093/cercor/bhm128 17652468 PMC2601025

[B33] Rushworth M. F. S. Behrens T. E. J. Johansen-Berg H. (2006). Connection patterns distinguish 3 regions of human parietal cortex. *Cereb. Cortex* 16 1418–1430. 10.1093/cercor/bhj079 16306320

[B34] Saalmann Y. B. Pigarev I. N. Vidyasagar T. R. (2007). Neural mechanisms of visual attention: how top-down feedback highlights relevant locations. *Science* 316 1612–1615. 10.1126/science.1139140 17569863

[B35] Saalmann Y. B. Pinsk M. A. Wang L. Li X. Kastner S. (2012). The pulvinar regulates information transmissions between cortical areas based on attention demands. *Science* 337 753–756. 10.1126/science.1223082 22879517 PMC3714098

[B36] Sack A. T. Kadosh R. C. Schuhmann T. Moerel M. Walsh V. Goebel R. (2008). Optimizing functional accuracy of TMS in cognitive studies: a comparison of methods. *J. Cogn. Neurosci.* 21 207–221. 10.1162/jocn.2009.21126 18823235

[B37] Sack A. T. Kohler A. Linden D. E. J. Goebel R. Muckli L. (2006). The temporal characteristics of motion processing in hMT/V5+: combining fMRI and neuronavigated TMS. *Neuroimage* 29 1326–1335. 10.1016/j.neuroimage.2005.08.027 16185899

[B38] Sandrini M. Umilta C. Rusconi E. (2011). The use of transcranial magnetic stimulation in cognitive neuroscience: a new synthesis of methodological issues. *Neurosci. Biobehav. Rev.* 35 516–536. 10.1016/j.neubiorev.2010.06.005 20599555

[B39] Scharnowski F. Rees G. Walsh V. (2013). Time and the brain: neurorelativity: the chronoarchitecture of the brain from the neuronal rather than the observer’s perspective. *Trends Cogn. Sci.* 17 51–52. 10.1016/j.tics.2012.12.005 23318094

[B40] Scolari M. Seidl-Rathkopf K. N. Kastner S. (2015). Functions of the human frontoparietal attention network: evidence from neuroimaging. *Curr. Opin. Behav. Sci.* 1 32–39. 10.1016/j.cobeha.2014.08.003 27398396 PMC4936532

[B41] Sestieri C. Pizzella V. Cianflone F. Romani G. L. Corbetta M. (2008). Sequential activation of human oculomotor centers during planning of visually-guided eye movements: a combined fMRI-MEG study. *Front. Hum. Neurosci.* 1:1. 10.3389/neuro.09.001.2007 18958215 PMC2525985

[B42] Sincich L. C. Park K. F. Wohlgemuth M. J. Horton J. C. (2004). Bypassing V1: a direct geniculate input to area MT. *Nat. Neurosci.* 7 1123–1128. 10.1038/nn1318 15378066

[B43] Stevens L. K. McGraw P. V. Ledgeway T. Schluppeck D. (2009). Temporal characteristics of global motion processing revealed by transcranial magnetic stimulation. *Eur. J. Neurosci.* 30 2415–2426. 10.1111/j.1460-9568.2009.07034.x 20092583

[B44] Tabachnick B. G. Fidell L. S. (2014). *Using Multivariate Statistics Pearson New International Edition.* Harlow: Pearson Education.

[B45] Talairach J. Tournoux P. (1988). *Co-Planar Stereotaxic Atlas of the Human Brain.* New York, NY: Thieme Medical Publishers.

[B46] Thier P. Andersen R. A. (1998). Electrical microstimulation distinguishes distinct saccade-related areas in the posterior parietal cortex. *J. Neurophysiol.* 80 1713–1735. 9772234 10.1152/jn.1998.80.4.1713

[B47] Tootell R. B. Reppas J. B. Kwong K. K. Malach R. Born R. T. Brady T. J. (1995). Functional analysis of human MT and related visual cortical areas using magnetic resonance imaging. *J. Neurosci.* 15 3215–3230. 7722658 10.1523/JNEUROSCI.15-04-03215.1995 PMC6577785

[B48] Van Essen D. C. Anderson C. H. Felleman D. J. (1992). Information processing in the primate visual system: an integrated systems perspective. *Science* 255 419–423. 10.1126/science.1734518 1734518

[B49] Van Essen D. C. Maunsell J. H. R. (1983). Hierarchical organization and functional streams in the visual cortex. *Trends Neurosci.* 6 370–375. 10.1016/0166-2236(83)90167-4

[B50] Walsh V. Ellison A. Battelli L. Cowey A. (1998). Task-specific impairments and enhancements induced by magnetic stimulation of human visual area V5. *Proc. R. Soc. Lond. B Biol. Sci.* 265 537–543. 10.1098/rspb.1998.0328 9569672 PMC1688918

[B51] Wang H. Crewther S. G. Liang M. Laycock R. Yu T. Alexander B. (2017). Impaired activation of visual attention network for motion salience is accompanied by reduced functional connectivity between frontal eye fields and visual cortex in strabismic amblyopia. *Front. Hum. Neurosci.* 11:195. 10.3389/fnhum.2017.00195 28484381 PMC5399630

[B52] Watson J. D. G. Myers R. Frackowiak R. S. J. Hajnal J. V. Woods R. P. Mazziotta J. C. (1993). Area V5 of the human brain: evidence from a combined study using positron emission tomography and magnetic resonance imaging. *Cereb. Cortex* 3 79–94. 10.1093/cercor/3.2.79 8490322

