# Supplementary material for: An fMRI-Neuronavigated Chronometric TMS Investigation of V5 and Intraparietal Cortex in Motion Driven Attention
Source: Front Hum Neurosci. 2018 Jan 4;11:638. doi: 10.3389/fnhum.2017.00638 (PMC5758491; doi:10.3389/fnhum.2017.00638)
Supplement: Supplementary file 1 [file Data_Sheet_1.pdf]

Supplementary material

Table S1. Talairach Coordinates of Peak Voxels From All Positive Clusters Activated for the Motion-Driven Attention Task, and Corresponding Structures

Cluster	Talairach Coord.			Cluster size	*t* value	R/L	Structure	Brodmann Area
	x	y	z					
1	44	-68	6	2292	6.57	R	Middle Occipital Gyrus	BA 37, 19
							Middle Temporal Gyrus	BA 37
2	32	25	0	1097	7.01	R	Inferior Frontal Gyrus	BA 47
							Insula	BA 13, 47
3	11	-74	45	3399	6.76	R	Precuneus	BA 7
4	23	-11	57	287	5.03	R	Middle Frontal Gyrus	BA 6
5	2	1	51	249	5.31	R	Medial Frontal Gyrus	BA 6
							Superior Frontal Gyrus	
6	2	22	36	341	5.95	R	Cingulate Gyrus	BA 32
							Medial Frontal Gyrus	
7	-28	-80	15	2045	6.04	L	Middle Occipital Gyrus	
							Cuneus	
8	-19	-11	51	202	4.18	L	Medial Frontal Gyrus	
9	-34	-38	39	354	5.89	L	Inferior Parietal Lobule	BA 40
10	-34	19	-3	767	6.38	L	Inferior Frontal Gyrus	BA 47
							Insula	BA 13, 47
12	-49	-74	0	767	7.26	L	Inferior Temporal Gyrus	BA 19
							Middle Occipital Gyrus	BA 19
							Inferior Temporal Gyrus	BA 18, 37

Table S2. Talairach Coordinates of Peak Voxels From Positive Clusters Activated for the Saccade Task, and Corresponding Structures

Cluster	Talairach Coord. X	Y	Z	Cluster size (1mm isotropic voxels)	*t* value	L/R	Structure	Brodmann Area
1	65	-38	15	219	6.69	R	Superior Temporal Gyrus	BA 22
2	53	-20	24	519	6.44	R	Postcentral Gyrus	
						R	Inferior Parietal Lobule	
3	47	10	33	3504	15.46	R	Middle Frontal Gyrus	BA 9
						R	Inferior Frontal Gyrus	BA 9
4	47	-62	-18	1317	7.20	R	Cerebellum: Declive	
						R	Fusiform Gyrus	BA 37
5	26	-44	42	962	7.39	R	Precuneus	
6	35	-47	-24	384	6.68	R	Cerebellum: Culmen	
8	17	-71	-18	15371	12.01	R	Cerebellum: Declive	
9	23	-65	45	2653	11.18	R	Superior Parietal Lobule	BA 7
						R	Precuneus	BA 7
11	23	1	6	467	11.77	R	Lentiform Nucleus: Putamen	
12	14	-2	15	225	5.33	R	Caudate: Caudate Body	
						R	Thalamus: Ventral Anterior Nucleus	
13	-1	-8	63	928	7.80	L	Medial Frontal Gyrus	BA 6
						L	Superior Frontal Gyrus	
14	2	1	51	365	7.03	R	Medial Frontal Gyrus	BA 6
						L	Medial Frontal Gyrus	
						R	Superior Frontal Gyrus	
17	-16	-53	0	489		L	Lingual Gyrus	BA 19
18	-22	-14	48	268	5.39	L	Sub-Gyral	
						L	Precentral Gyrus	
19	-28	-92	15	366	7.43	L	Middle Occipital Gyrus	BA 18,19
20	-37	-68	-12	340	8.36	L	Fusiform Gyrus	BA 19
						L	Middle Occipital Gyrus	
21	-46	-8	51	726		L	Precentral Gyrus	BA 4, 6
22	-43	-74	-6	237	5.01	L	Inferior Occipital Gyrus	BA 19
						L	Middle Occipital Gyrus	BA 18, 19
23	-52	-2	39	701	6.14	L	Precentral Gyrus	BA 6

D1: Definition of TMS stimulation sites per individual

To define ROIs per individual, a posterior right hemisphere segment of the Talairach brain mask was created (i.e., including y = 0 to -127, and x = 0 to 127). Cluster threshold estimation was performed on the uninterpolated masked *t*-map at $\alpha < .05$, returning minimum cluster size $k = 6$ voxels. To construct a V5 mask, the *t*-map was subjected to cluster extent thresholding using the modified Talairach mask. At $\alpha < .05$, this specified $k = 5$.

For the motion-driven attention task, individual analyses were performed using a GLM and a contrast subtracting the baseline from active condition, thresholded using a False Discovery Rate of $q < .02$. For the V5 task, individual analyses were also performed, using a threshold in the least conservative case of $p < .05$ and cluster extent thresholding to correct for multiple comparisons. In most cases of extensive activity, this threshold was increased to a more stringent level to achieve better separation of functional clusters and hence determine relevant maxima per individual.

As discussed by Sack et al. (2008), there was substantial variability in the location of activation maxima and surrounding activity per participant. Some participants showed discrete clusters with maxima outside of the group clusters, some had more highly activated maxima contralaterally, and some had extensive activity spreading to other local maxima. Selection of coordinates was informed by three factors: individual activation clusters and their maxima, group Region Of Interest locations, and individual anatomy with relation to the functional locations established in the literature. The decision process was as follows: if there was a clear individual activation maximum within the group-masked ROI or nearby, this was chosen. For disperse or, e.g., contralateral activity, the most highly activated voxel within the group-masked region was chosen. As a third option, points were chosen that were active within the group-masked ROI and were most anatomically appropriate for that individual. The mean (SD in brackets) Talairach coordinates (x, y, z) stimulated were: mIPS: 26.6(2.0), -60.1(2.4), 51.7(4.6); pIPS: 14.7(4.2), -77.7(3.8), 42.3(4.1); V5: 45.2(4.7), -69.6(2.4), 2.4(3.7). These points are illustrated in Figure 2b, and listed per individual in Table S3.

Table S3. Talairach Coordinates of Areas Stimulated Using TMS per Participant and Criteria Used

Site	mIPS				pIPS				V5			
Tal.	x	y	z		x	y	z		x	y	z	
Partic.												
P01	27	-57	52	*c*	14	-73	48	*b*	48	-66	1	*b*
P02	29	-59	51	*a*	17	-79	42	*b*	48	-70	7	*c*
P03	26	-65	47	*b*	20	-83	39	*a*	49	-70	-2	*b*
P04	23	-59	54	*a*	5	-75	48	*c*	41	-71	2	*b*
P05	26	-62	42	*a*	13	-83	36	*b*	50	-65	-4	*b*
P08	26	-59	54	*c*	15	-75	42	*c*	47	-71	6	*b*
P09	30	-59	54	*c*	16	-80	42	*c*	36	-71	6	*b*
P10	26	-59	57	*c*	18	-77	45	*b*	41	-71	3	*b*
P11	26	-62	54	*a*	14	-74	39	*b*	47	-71	3	*b*
Average	26.6	-60.1	51.7		14.7	-77.7	42.3		45.2	-69.6	2.4	
Std. Dev.	2.0	2.4	4.6		4.2	3.8	4.1		4.7	2.4	3.7	

[a] Based on individual local activity peak falling outside of the group masked area [b] Individual maximum constrained to within group masked area [c] Area in the intersection of the individual and group masked activity. Tal. = Talairach axis, mIPS = middle intraparietal sulcus, pIPS* = posterior intraparietal sulcus

D2: Protocol for TMS coregistration, intensity level establishment, and neuronavigation

Coregistration of the head and structural scan was performed. Ultrasound transmitters were affixed above the eyebrows, and on the tip of the nose. Fiducial points were defined on the scalp above the tip of each ear and on the nasion. Corresponding points on the participant were indicated with the digitiser pen, repeated until coregistration was within 2 mm. The coil was coregistered, and angle and proximity to the scalp verified. The cortical surface reconstruction was used for navigation of structural landmarks. 1 mm^3 surface meshes of point coordinates were used as TMS targets, and individual clusters of activity used to visually aid neuronavigation. Participants were given 30 dB sound-reducing earplugs.
TMS intensity levels were established for the first site, starting with pulses at 30% stimulator output, then in 5 % increments to 60 % if the participant indicated comfort. If the participant indicated discomfort or muscle twitches, or if blinking occurred, intensity was lowered. Parietal stimulation was mostly free of such issues. For V5, muscle twitches and discomfort were indicated by almost all participants, upon which the intensity was lowered. The average intensity for V5 TMS (45.56 %) was markedly lower than for parietal areas (57.89 % for mIPS, 59.11 % for pIPS). For V5, in some cases where minor muscle twitches occurred even at low stimulation intensities (presumably due to proximity of facial nerves), participants voluntarily indicated willingness to continue. Thus, some blocks of V5 TMS were conducted in the presence of minor twitches.

The TMS blocks were then run. The coil position was adjusted manually, with the aim of keeping the center of the induced field within 5 mm of the target, and minimising the coil-to-target distance. Between blocks, the coil was changed and coregistered.

Table S4. Two-Tailed, Single Sample T-Tests Comparing Accuracy for Each SOA With Zero, for mIPS TMS

SOA (ms)	*t*	*df*	*p*	*d*	Mean Difference from 0	95% Confidence Interval of the Difference	
						Lower	Upper
mIPS_0	0.030	8	.977	0.010	0.009	-0.704	0.723
mIPS_30	-1.471	8	.180	0.490	-0.531	-1.363	0.302
mIPS_60	-1.483	8	.176	0.494	-0.349	-0.891	0.194
mIPS_90	0.819	8	.437	0.273	0.256	-0.465	0.978
mIPS_120	1.342	8	.216	0.447	0.386	-0.277	1.049
mIPS_150	-2.140	8	.065	0.713	-0.551	-1.145	0.043
mIPS_180	4.452	8	.002	1.484	0.779	**0.376**	**1.183**

Reaction time results.

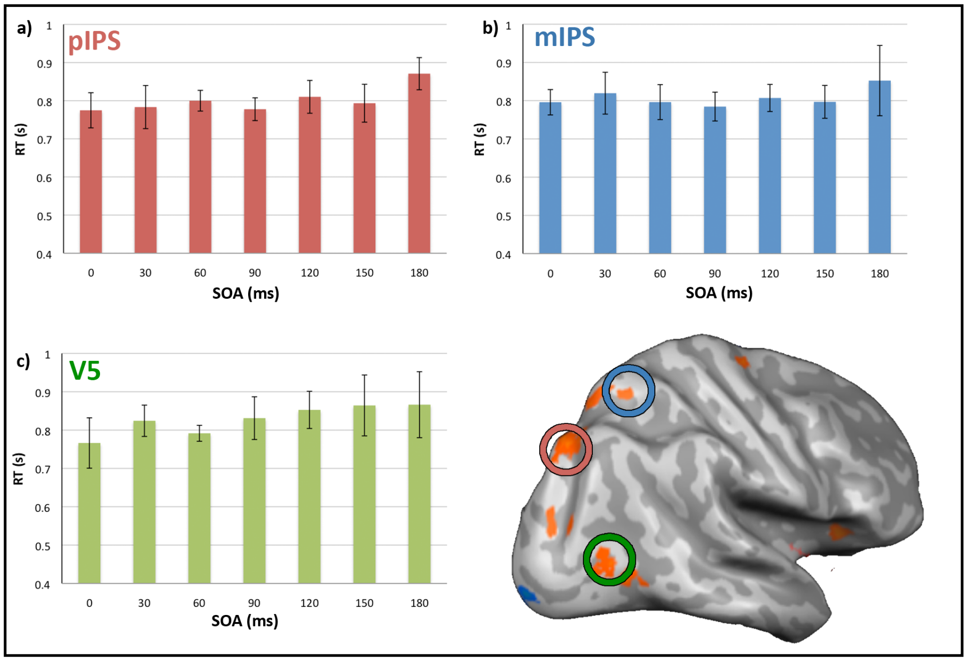

Figure S1. Reaction time results for each site of stimulation. Y axes are accuracy z-scores, x axes are SOA between 0-180 ms. Error bars are 95% confidence intervals of the mean, calculated as per Morey (2008). These confidence intervals allow inference of difference in means across SOAs. Right hemisphere surface shown with group activity for the Motion-Driven Attention task, and mIPS, pIPS and V5 TMS application sites circled.

One-way repeated measures ANOVAs were performed on RT measures for each site of TMS application, and results are listed in Table S6.

***Table S5.* One-Way ANOVA Results for RT for each site**

Site	*df*	*F*	*p*	η^2
V5	(6,48)	2.072	.074	.206
mIPS	(3,28)	0.993	.419	.110
pIPS	(6,48)	3.119	.012	.280

* Indicates *p* values lower than .100.

D3: Linear and nonlinear trend analysis

Trend analysis is used for RT to investigate the overall shape of the data (Howell, 2010) and whether there were statistically significant systematic trends across variables. Also, to avoid the high number of multiple comparisons that would have been incurred by using post-hoc *t*-tests.

ANOVA results revealed main effects of SOA for RT for V5 and pIPS. These effects appear to reflect increased reaction times, particularly at the 180 ms SOA, with regards to other SOAs, and possibly a comparative decrease in RT at the 0 ms SOA. Trend analysis was employed to determine whether the increase in RT across SOAs could be best described as a significant linear or nonlinear trend.

For V5 stimulation, RT showed a significant linear trend, $F(1,8) = 6.357$, $p = .036$, $\eta^2 = .443$. For RT for mIPS stimulation, there were no significant trends. For pIPS stimulation, there was a strong linear trend, $F(1,8) = 12.276$, $p = .008$, $\eta^2 = .605$, as well as quadratic, $F(1,8) = 5.117$, $p = .054$, $\eta^2 = .390$, and near-significant sextic trends, $F(1,8)$ 4.475= , $p = .067$, $\eta^2 = .359$.

Table S6. **Tests of normality for TMS accuracy and RT data**

Condition	Accuracy			Reaction Time		
	Shapiro-Wilk statistic	*df*	*p*	Shapiro-Wilk statistic	*df*	*p*
mIPS_0	.951	9	.702	.856	5	.215
mIPS_30	.877	9	.146	.873	5	.278
mIPS_60	.893	9	.213	.913	5	.486
mIPS_90	.952	9	.717	.947	5	.716
mIPS_120	.917	9	.367	.940	5	.665
mIPS_150	.982	9	.974	.871	5	.272
mIPS_180	.951	9	.701	.968	5	.864
pIPS_0	.876	9	.141	.959	5	.799
pIPS_30	.959	9	.784	.915	5	.500
pIPS_60	.889	9	.195	.864	5	.243
pIPS_90	.872	9	.128	.926	5	.569
pIPS_120	.897	9	.237	.969	5	.872
pIPS_150	.952	9	.716	.885	5	.332
pIPS_180	.938	9	.561	.905	5	.436
V5_0	.879	9	.155	.651	5	.003
V5_30	.947	9	.653	.910	5	.467
V5_60	.934	9	.525	.932	5	.612
V5_90	.895	9	.222	.853	5	.205
V5_120	.968	9	.878	.974	5	.898
V5_150	.948	9	.668	.896	5	.387

V5_180	.946	9	.650	.935	5	.629

***Table S7.* Z-skew and z-kurtosis scores for RT and accuracy data**

	Reaction time		Accuracy	
Condition	z-skew	z-kurtosis	z-skew	z-kurtosis
mIPS_0ms	0.540	-0.226	-0.737	0.772
mIPS_30ms	1.060	0.597	-0.126	-1.397
mIPS_60ms	0.617	-0.497	-1.079	-0.441
mIPS_90ms	0.335	0.039	-0.928	-0.168
mIPS_120ms	-0.256	-0.699	0.258	-1.255
mIPS_150ms	1.035	-0.116	-0.504	-0.195
mIPS_180ms	-1.183	0.694	-0.189	-0.541
pIPS_0ms	0.445	-0.371	0.522	-1.147
pIPS_30ms	-0.470	-0.613	-0.075	-0.548
pIPS_60ms	-0.378	-0.391	1.594	0.923
pIPS_90ms	0.218	-0.457	1.108	0.009
pIPS_120ms	-1.157	0.457	-0.993	0.010
pIPS_150ms	1.509	1.148	-0.592	-0.492
pIPS_180ms	-1.134	-0.507	0.379	-0.957
V5_0ms	0.110	-0.037	-0.231	-1.445
V5_30ms	1.490	0.548	-0.156	-0.744
V5_60ms	1.049	0.057	-0.379	-0.962
V5_90ms	0.260	-0.021	-1.076	0.751
V5_120ms	1.403	0.901	-0.068	-0.274
V5_150ms	0.138	-0.705	0.399	-0.043
V5_180ms	-0.701	0.434	0.401	-0.472

References

Howell, D. C. (2010). *Statistical Methods for Psychology* (7th ed.). Belmont, CA: Cengage Wadsworth.

Morey, R. D. (2008). Confidence intervals from normalized data: A correction to Cousineau (2005). *reason, 4*(2), 61-64.
