# Supplementary material for: An fMRI-Neuronavigated Chronometric TMS Investigation of V5 and Intraparietal Cortex in Motion Driven Attention
Source: Front Hum Neurosci. 2018 Jan 4;11:638. doi: 10.3389/fnhum.2017.00638 (PMC5758491; doi:10.3389/fnhum.2017.00638)
Supplement: Supplementary file 4 [file Data_Sheet_4.DOCX]

## (Paste into R script. Journal submission system didn’t allow upload of scripts)

#### Make Morey (2008) confidence intervals for RT data

library(reshape2)

## import

mips.rt <- read.csv(file="RT_mIPS_for_Morey_CIs.csv", head=TRUE, sep=",", na.strings="")

pips.rt <- read.csv(file="RT_pIPS_for_Morey_CIs.csv", head=TRUE, sep=",", na.strings="")

v5.rt <- read.csv(file="RT_V5_for_Morey_CIs.csv", head=TRUE, sep=",", na.strings="")

## make sure partic ID is a factor

mips.rt$partic <- factor(mips.rt$partic)

pips.rt$partic <- factor(pips.rt$partic)

v5.rt$partic <- factor(v5.rt$partic)

## convert data into long format

mips.rt.long <- melt(mips.rt, id.vars="partic",

measure.vars= c("mIPS_0ms_RT", "mIPS_30ms_RT", "mIPS_60ms_RT", "mIPS_90ms_RT", "mIPS_120ms_RT", "mIPS_150ms_RT","mIPS_180ms_RT"),

variable.name = "condition")

pips.rt.long <- melt(pips.rt, id.vars="partic",

measure.vars= c("pIPS_0ms_RT", "pIPS_30ms_RT", "pIPS_60ms_RT", "pIPS_90ms_RT", "pIPS_120ms_RT", "pIPS_150ms_RT","pIPS_180ms_RT"),

variable.name = "condition")

v5.rt.long <- melt(v5.rt, id.vars="partic",

measure.vars= c("V5_0ms_RT", "V5_30ms_RT", "V5_60ms_RT", "V5_90ms_RT", "V5_120ms_RT", "V5_150ms_RT","V5_180ms_RT"),

variable.name = "condition")

## HELPER FUNCTIONS-------------------

## This section helper functions and comments copied verbatim directly from here: http://www.cookbook-r.com/Graphs/Plotting_means_and_error_bars_(ggplot2)/#Helper%20functions

## Gives count, mean, standard deviation, standard error of the mean, and confidence interval (default 95%).

## data: a data frame.

## measurevar: the name of a column that contains the variable to be summariezed

## groupvars: a vector containing names of columns that contain grouping variables

## na.rm: a boolean that indicates whether to ignore NA's

## conf.interval: the percent range of the confidence interval (default is 95%)

summarySE <- function(data=NULL, measurevar, groupvars=NULL, na.rm=FALSE,

conf.interval=.95, .drop=TRUE) {

library(plyr)

# New version of length which can handle NA's: if na.rm==T, don't count them

length2 <- function (x, na.rm=FALSE) {

if (na.rm) sum(!is.na(x))

else length(x)

}

# This does the summary. For each group's data frame, return a vector with

# N, mean, and sd

datac <- ddply(data, groupvars, .drop=.drop,

.fun = function(xx, col) {

c(N = length2(xx[[col]], na.rm=na.rm),

mean = mean (xx[[col]], na.rm=na.rm),

sd = sd (xx[[col]], na.rm=na.rm)

)

},

measurevar

)

# Rename the "mean" column

datac <- rename(datac, c("mean" = measurevar))

datac$se <- datac$sd / sqrt(datac$N) # Calculate standard error of the mean

# Confidence interval multiplier for standard error

# Calculate t-statistic for confidence interval:

# e.g., if conf.interval is .95, use .975 (above/below), and use df=N-1

ciMult <- qt(conf.interval/2 + .5, datac$N-1)

datac$ci <- datac$se * ciMult

return(datac)

}

## Norms the data within specified groups in a data frame; it normalizes each

## subject (identified by idvar) so that they have the same mean, within each group

## specified by betweenvars.

## data: a data frame.

## idvar: the name of a column that identifies each subject (or matched subjects)

## measurevar: the name of a column that contains the variable to be summariezed

## betweenvars: a vector containing names of columns that are between-subjects variables

## na.rm: a boolean that indicates whether to ignore NA's

normDataWithin <- function(data=NULL, idvar, measurevar, betweenvars=NULL,

na.rm=FALSE, .drop=TRUE) {

library(plyr)

# Measure var on left, idvar + between vars on right of formula.

data.subjMean <- ddply(data, c(idvar, betweenvars), .drop=.drop,

.fun = function(xx, col, na.rm) {

c(subjMean = mean(xx[,col], na.rm=na.rm))

},

measurevar,

na.rm

)

# Put the subject means with original data

data <- merge(data, data.subjMean)

# Get the normalized data in a new column

measureNormedVar <- paste(measurevar, "_norm", sep="")

data[,measureNormedVar] <- data[,measurevar] - data[,"subjMean"] +

mean(data[,measurevar], na.rm=na.rm)

# Remove this subject mean column

data$subjMean <- NULL

return(data)

}

## Summarizes data, handling within-subjects variables by removing inter-subject variability.

## It will still work if there are no within-S variables.

## Gives count, un-normed mean, normed mean (with same between-group mean),

## standard deviation, standard error of the mean, and confidence interval.

## If there are within-subject variables, calculate adjusted values using method from Morey (2008).

## data: a data frame.

## measurevar: the name of a column that contains the variable to be summariezed

## betweenvars: a vector containing names of columns that are between-subjects variables

## withinvars: a vector containing names of columns that are within-subjects variables

## idvar: the name of a column that identifies each subject (or matched subjects)

## na.rm: a boolean that indicates whether to ignore NA's

## conf.interval: the percent range of the confidence interval (default is 95%)

summarySEwithin <- function(data=NULL, measurevar, betweenvars=NULL, withinvars=NULL,

idvar=NULL, na.rm=FALSE, conf.interval=.95, .drop=TRUE) {

# Ensure that the betweenvars and withinvars are factors

factorvars <- vapply(data[, c(betweenvars, withinvars), drop=FALSE],

FUN=is.factor, FUN.VALUE=logical(1))

if (!all(factorvars)) {

nonfactorvars <- names(factorvars)[!factorvars]

message("Automatically converting the following non-factors to factors: ",

paste(nonfactorvars, collapse = ", "))

data[nonfactorvars] <- lapply(data[nonfactorvars], factor)

}

# Get the means from the un-normed data

datac <- summarySE(data, measurevar, groupvars=c(betweenvars, withinvars),

na.rm=na.rm, conf.interval=conf.interval, .drop=.drop)

# Drop all the unused columns (these will be calculated with normed data)

datac$sd <- NULL

datac$se <- NULL

datac$ci <- NULL

# Norm each subject's data

ndata <- normDataWithin(data, idvar, measurevar, betweenvars, na.rm, .drop=.drop)

# This is the name of the new column

measurevar_n <- paste(measurevar, "_norm", sep="")

# Collapse the normed data - now we can treat between and within vars the same

ndatac <- summarySE(ndata, measurevar_n, groupvars=c(betweenvars, withinvars),

na.rm=na.rm, conf.interval=conf.interval, .drop=.drop)

# Apply correction from Morey (2008) to the standard error and confidence interval

# Get the product of the number of conditions of within-S variables

nWithinGroups <- prod(vapply(ndatac[,withinvars, drop=FALSE], FUN=nlevels,

FUN.VALUE=numeric(1)))

correctionFactor <- sqrt( nWithinGroups / (nWithinGroups-1) )

# Apply the correction factor

ndatac$sd <- ndatac$sd * correctionFactor

ndatac$se <- ndatac$se * correctionFactor

ndatac$ci <- ndatac$ci * correctionFactor

# Combine the un-normed means with the normed results

merge(datac, ndatac)

}

## Use summarySEwithin function to get CIs -------------

mips.rt.ci <- summarySEwithin(mips.rt.long, measurevar="value", withinvars="condition",

idvar="partic", na.rm=FALSE, conf.interval=.95)

pips.rt.ci <- summarySEwithin(pips.rt.long, measurevar="value", withinvars="condition",

idvar="partic", na.rm=FALSE, conf.interval=.95)

v5.rt.ci <- summarySEwithin(v5.rt.long, measurevar="value", withinvars="condition",

idvar="partic", na.rm=FALSE, conf.interval=.95)

## reorder the rows

mips.rt.ci <- mips.rt.ci[c(1,5,6,7,2,3,4),]

pips.rt.ci <- pips.rt.ci[c(1,5,6,7,2,3,4),]

v5.rt.ci <- v5.rt.ci[c(1,5,6,7,2,3,4),]

## concatenate dfs

all.rt.ci <- rbind(mips.rt.ci, pips.rt.ci, v5.rt.ci)

## export

write.table(all.rt.ci, file="all_morey_cis.csv", sep=",", row.names=FALSE)
